# Tracheobronchopathia osteochondroplastica associated with skin cancer: a case report and review of the literature

**DOI:** 10.1186/1756-0500-7-637

**Published:** 2014-09-12

**Authors:** Mustapha Laine, Sanaa Elfihri, Fouad Kettani, Jamal Eddine Bourkadi

**Affiliations:** Departement of Pneumology, Moulay Youssef University Hospital Center, Faculty of Medicine and Pharmacy, Mohamed V University, 23 rue Belgrade, App 1, Ocean Rabat, Morocco; Institute of Pathology Nations Unies, Rabat, Morocco

**Keywords:** Tracheobronchopathia osteochondroplastica, Trachea, Fiberoptic bronchoscopy

## Abstract

**Background:**

Tracheobronchopathia osteochondroplastica (TO) is a rare disorder of unknown cause affecting the large airways. It is characterized by the accumulation of bony and cartilaginous nodules in the tracheal and bronchial mucosa.

Approximately 300 cases of tracheobronchopathia osteochondroplastica have been reported since Wilks first identified it in 1857. Tomography and computed tomography scanning (CT) can be suggestive but final verification requires biopsy.

Neoplastic disorders are, among others, blamed in etiology. We describe here, for the first time, a case of TO associated with skin cancer.

**Case presentation:**

A 40-year-old man with a scalp cancer was admitted for further evaluation of an occasional dry cough. Her medical history was otherwise unremarkable, and physical examination showed no abnormalities.

The chest CT scan demonstrated multiple nodular densities in the trachea and proximal bronchi. The fiberoptic bronchoscopy showed multiple nodules in the trachea suggesting a malignant infiltration.

Microscopic examination of the biopsy material revealed fragments of normal cartilage and bone formation with normal mucosa which confirmed the diagnosis of TO.

Patient underwent surgery for scalp cancer. For TO, case has followed up. At twelve-month follow up, scalp tumor did not recur and cough ceased.

**Conclusion:**

TO is a rare, benign disease that should be kept in mind especially in patients with tracheal irregularities in their chest imaging. Association with malignant tumors is reported.

In patients with malignancy, TO can easily be misdiagnosed if it is not known as a diagnosis possibility of malignant invasion of the trachea. Therefore, it is important to be aware of this possibility, in order to prevent unnecessary treatments to patients.

## Background

Tracheobronchopathia osteochondroplastica (TO) is a rare benign disorder that affects the tracheobronchial tree. It is characterized by the presence of multiple osseous and/or cartilaginous submucosal nodules protruding into the lumen of the trachea and large bronchi [1, 2]. TO was first described in detail by Wilks in 1857 [3]. Since then, approximately 300 cases have been reported [1]. Its etiology remains unknown; chronic inflammatory processes or degeneration, metabolic and neoplastic disorders are blamed in the etiology [4].

The bronchoscopic and computed tomography appearance of TO are highly suggestive. However, pathologic examination of bronchial biopsy is needed to make a definitive diagnosis. We report a case of TO associated with skin cancer. To our knowledge, this association has never been previously reported.

## Case presentation

**Figure 1:**
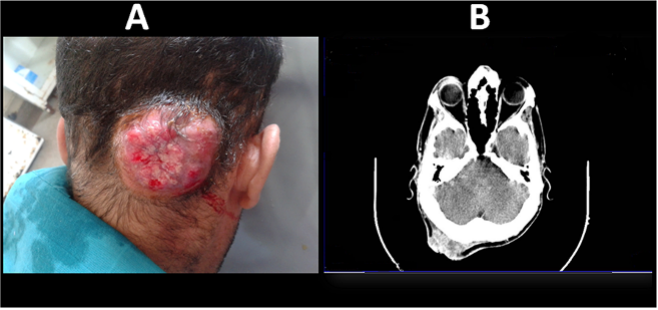
**Skin carcinoma: Macroscopic view (A), and cranial computed tomography scan (B).** Exophytic lesion of the scalp.

**Figure 2:**
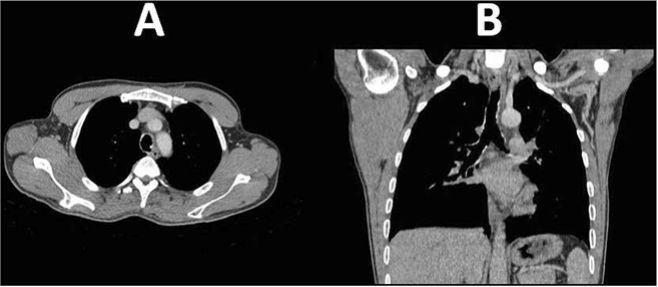
**Chest computed tomography scan: Axial sections (A) and coronal reconstruction (B) showing thickening and calcified lesions of the trachea.**

A 40-year-old Moroccan man, recently diagnosed with a scalp cancer (Figure 1) presented with occasional dry cough. He was non smoker and had no history of tuberculosis or bronchial asthma. Apart the skin tumor, physical examination showed no abnormalities. There was no clubbing or lymphadenopathy. His past medical history was unremarkable. The Chest radiography was normal. Chest computed tomography (Figure 2) showed diffuse, irregular nodules and calcification of the tracheal wall and proximal bronchi. To evaluate the nature of the tracheal lesions, fiberoptic bronchoscopy was necessary. However, it was difficult to convince patient to undergo bronchoscopy for an occasional cough.

**Figure 3:**
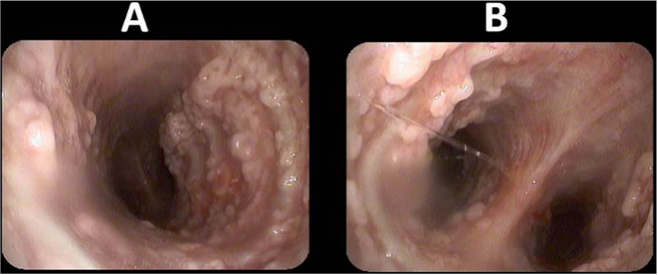
**Bronchoscopic view showed multiple, irregular nodules in the trachea (A) and the main bronchi (B), sparing the posterior wall.**

**Figure 4:**
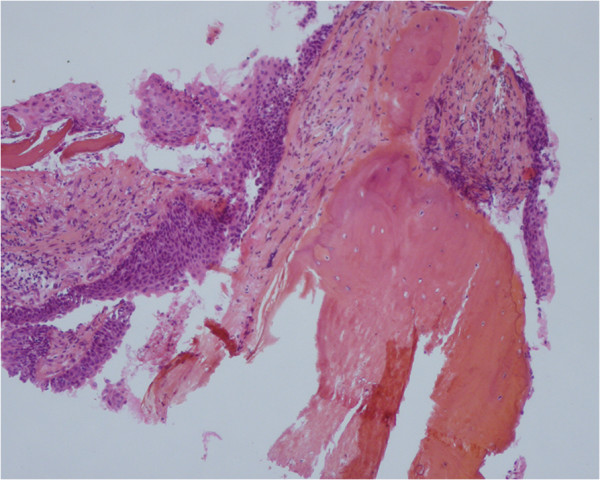
**Pathological examination of specimen’s biopsy showed squamous metaplasia with ectopic bony tissue under the respiratory epithelium.** (Haematoxylin, eosin, and safran × 400).

Bronchoscopy (Figure 3), revealed multiple, irregular nodules protruding into the tracheal lumen, extending through the right and left main bronchi. The posterior tracheal membrane was spared. Because of the skin cancer, we initially expected these lesions to be malignant. Various biopsies were taken. Microscopic analysis of specimens showed squamous metaplasia with ectopic bony tissue (Figure 4), consistent with the diagnosis of TO. No malignant cells were seen.

The case was considered TO associated with skin cancer.

Scalp tumor staging procedure, including bone scintigraphy, chest-abdomen and brain CT, did not detect distant metastasis. The case was classified stage 2 of TNM classification.

Patient underwent surgery for scalp cancer. For TO, he has followed up. Any special treatment was not performed. At twelve-month follow up, scalp tumor did not recur and cough ceased. Endoscopic follow up confirmed that the endotracheal and bronchial lesions were not progressive.

A timeline in the form of a table gives the specific dates of important components of the case (Table 1).

**Table 1:** **Timeline**

08.1973	Birth
	- Non significant medical history
	- Non smoker
11.2012	Occasional dry cough
04.2013	Appearance of a scalp tumor
08.2013	- Biopsy of the scalp tumor: Squamous cell carcinoma
	- Tumor staging:
	• Brain CT: no evidence of regional metastasis or involved lymph nodes.
	• Chest CT: multiple nodular densities in the trachea and proximal bronchi
	• Abdomen CT: normal
	• Bone scintigraphy: normal
09.2013	- Bronchoscopy:
	• Multiple nodules protruding into the tracheal lumen
	• Biopsies: TO diagnosis
	- No treatment performed
	- Follow-up recommended
10.2013	Surgery for scalp cancer
08.2014	Twelve-month follow-up
	- TO:
	• Clinical assessment: cough ceased
	• Bronchoscopy: no progression of the lesions
	- Scalp cancer: No signs of recurrence

### Discussion

Tracheobronchopathia osteochondroplastica (TO) is a rare benign disorder characterized by numerous osseous and cartilaginous submucosal nodules projecting into the lumen of the trachea and bronchi. TO was originally described by Wilks in 1857. At autopsy, he found the larynx, trachea and bronchi to be covered by a number of bony plates [3].

Since then, around 300 cases have been reported in literature [1]. Most cases had been an incidental finding at autopsy, but an increasing number of cases were diagnosed during bronchoscopic examination [5] or chest computed tomography (CT) scanning [6]. TO is detected in approximately one of every 2000 bronchoscopies [7].

The nodules originate in the airway cartilages and thus typically spare the posterior membranous wall of the airways.

The disease is usually identified in adults older than 50 years, but it has also been described in children. There is no sex predominance and no clear link with smoking [8].

Clinically, TO is usually asymptomatic and incidentally discovered, but can present with wheezing, cough, hemoptysis, dyspnea, and rarely obstructive symptoms [9]. Few cases of TO are diagnosed after difficult intubation [8, 10]. Currently, diagnosis is made during bronchoscopy, however imaging studies, most frequently CT or magnetic resonance imaging, can identify TO [11]. Chest radiography is usually normal.

CT can reveal multiple nodular submucosal irregularities, some of which calcified, with sparing of the posterior tracheal wall. It is also important in detecting the complications such as lobar collapse and post obstructive bronchiectasis [12]. Detection and diagnosis of TO by a CT scan require careful interpretation of the findings with extensive knowledge of the disorder.

In our case, the chest radiography was normal, but the CT scan demonstrated multiple nodular densities in the trachea.

If CT scanning may be suggestive, final verification requires bronchoscopy with biopsy.

Endoscopic features are typical and pathognomonic. TO appears as whitish, hard spicules projecting into the tracheal lumen from the anterior and lateral walls, with sparing of the posterior wall. Also the larynx and the main bronchi could be involved [8]. The spiculs are firm and frequently difficult to biopsy through the fiberoptic bronchoscope. In our case, bronchoscopic findings were characteristic of TO.

Histological examination of the nodules is non-specific, it usually reveals varying combinations of cartilaginous, osseous and haemopoietic tissue within a calcified protein matrix, protruding into the bronchial lumen. The overlying mucosa is often the site of squamous metaplasia [12].

Lesions do not carry any malignant transformation potential. They progress slowly and rarely need treatment. In a long time period, obstruction and recurrent infections might occur [4].

There is no definitive therapy: treatment is only symptomatic, which includes antibiotics in case of bacterial infections, mechanical measures to remove obstructing nodules using either cryotherapy, laser excision, external beam irradiation, radiotherapy, stent insertion or surgical resection therapy [13–15].

The differential diagnosis to the bronchoscopic picture of multiple nodules includes TO, amyloidosis, endobronchial sarcoidosis, calcific tuberculosis, papillomatosis and tracheobronchial calcinosis [1].

The etiology of TO remains obscure. Hypotheses include acquired connective tissue metaplasia, cartilaginous ring exotoses, ossification of elastic cartilage, and reactive inflammation secondary to chronic infection. The potential for genetic predisposition has also been considered, given the rare occurrence of familial cases [7].

A few cases have been associated with lung cancer [16, 17], and a few with thyroid tumor or thymoma [18, 19].

In the Japanese literature, approximately 140 cases have been published since the first one reported by Kidokoro in 1938 [20].

Yokoyama [21], referring to japanese literature, reported that malignant tumors were associated among 19% of the patients with TO. He also reported that lung cancer was found in 11.1% of patients with this disease, and the most frequent histologic type among the concomitant lung cancer is adenocarcinoma. However, these findings are inconsistent and the association may be coincidental [1].

Our patient was diagnosed as TO and skin cancer. To our knowledge, skin cancer has never previously been reported in association with TO.

In our case, we didn’t consider TO initially given its rarity and the context of malignancy. The histological findings confirmed the diagnosis.

If TO is not suspected in the differential diagnosis of malignant invasion of the trachea, it can easily be misdiagnosed especially in the patients complicated with malignancy at the same time.

Therefore, in cases of TO complicated with cancer, careful examination is necessary, including bronchoscopy and biopsy of tracheal lesions.

## Conclusion

TO is a rare, benign disorder of tracheobronchial tree. Neoplastic disorders are, among others, blamed in the etiology. We reported here, for the first time, a case associated with skin cancer.

In patients with malignant tumors, TO can easily be misdiagnosed if it is not known as a diagnosis possibility of malignant invasion of the trachea. Therefore, it is important to be aware of this possibility, in order to prevent unnecessary treatments like chemotherapy to patients.

## Consent

Written informed consent was obtained from the patient for publication of this Case report and any accompanying images. A copy of the written informed consent is available for review by the Editor-in-Chief of this journal.
